# The need for clinical ethics consultation: a monocentric observational survey study in the intensive care unit (Consul.E.T.I. study)

**DOI:** 10.1186/s44158-022-00069-0

**Published:** 2022-09-14

**Authors:** Matteo Filippini, Federico Nicoli, Mario Picozzi, Nicola Latronico

**Affiliations:** 1 Department of Anesthesia, Intensive Care and Emergency, ASST Spedali Civili University Hospital, Piazzale Spedali Civili, 1, Brescia, 25123 Italy; 2 Center for Clinical Ethics, Department of Biotechnologies and Life Sciences, University of Insubria, Varese, Italy; 3 Clinical Ethics Service, Domus Salutis Clinic, Teresa Camplani Foundation, Brescia, Italy; 4 Department of Medical and Surgical Specialties, Radiological Sciences and Public Health, University of Brescia, Brescia, 25123 Italy

**Keywords:** Clinical ethics consultation, Clinical ethics service, Intensive care unit

## Abstract

**Background:**

The current organizational structure of the Italian healthcare system does not include the institutionalization of clinical ethics services.

To describe the need for structured clinical ethics consultation services for ICU staff members in the intensive care unit (ICU), a monocentric observational survey study was performed utilizing a paper-based questionnaire.

**Results:**

A total of 73 healthcare professionals (HCPs) responded out of a team of 84 people (87%). The results showed that the need for ethics consultation in the ICU is urgent, the institutionalization of the clinical ethics service is perceived as useful and should be a priority, and the issues on which the HCPs would like ethics consultation to focus are various and belong to “end of life” topics.

**Conclusions:**

HCPs believe that the clinical ethicist should become an integral part of ICU healthcare teams, offering consultations similar to the other specialistic consultations carried out in hospitals.

**Supplementary Information:**

The online version contains supplementary material available at 10.1186/s44158-022-00069-0.

## Introduction

Clinical ethics consultation (CEC) is one of the main functions of a clinical ethics service (CES). It allows professionals not only to analyze and resolve ethical dilemmas present in daily clinical practice but also to promote an ethical culture in healthcare settings and to promptly identify the issues that require planning of training courses for healthcare professionals (HCPs). CES should be an integral part of the care pathway as an ongoing support to clinical practice, since ethics questions arise in different content domains (e.g., shared decision making with patients; ethical practices in end-of-life and at beginning of life; patient privacy and confidentiality; professionalism in patient care; ethical practices in resource allocation, in business and management and in research) [1–7].

Every ethical question arises from the difficulty in balancing the different possible therapeutic options in given clinical situations with the different individual evaluations regarding the various feasible choices [8–14].

The clinical ethics consultant helps to define the criteria required in order to reach ethically justified and hopefully shared decisions between the HCPs and, where possible, with the patient and family members.

## Background

The “Clinical Ethics Consultation in the Intensive Care Unit” (Consulenza Etica in Terapia Intensiva, Consul.E.T.I.) study was developed in the context of a broader project launched at the Spedali Civili University Hospital of Brescia (Italy), regarding the introduction of the practice of clinical ethics consultation in complex hospital settings [15, 16], beginning with the intensive care unit (ICU) [17].

As the study was almost unprecedented in Italy, the project had a somewhat slow start and took an approach aimed at exploring the possible ways of practical implementation.

The first step was taken in the summer of 2019, with the introduction of a clinical ethicist who was present in the ward for 2 weeks; their task was to become familiar with the reality of the ICU and to initiate a dialogue with the HCPs. The next step was to interview the HCPs in order to identify their specific needs and to collect suggestions which could be useful to the project. In the period which followed, a more practical task was elaborated, the final objective of which was to establish the CES as an official position in ICUs.

## Methods

This monocentric and observational study was conducted from October 2020 to November 2020 at the ICU 2 of the Spedali Civili University Hospital of Brescia, Italy, which was a general ICU with 13 beds. Three of them were dedicated to post-operative elective patients treated for all surgical conditions, with the exception of cardiosurgical and solid organ transplantation. In 2020, 1255 patients were admitted (512 elective post-operative and 736 admitted for emergency conditions), and 138 of them died (11% of all patients, 19% of patients admitted for emergency conditions). The main reasons for admission except for elective post-operative patients were as follows: major trauma (552 patients), respiratory failure (452 patients), cardiocirculatory failure (226 patients), sepsis or septic shock (188 patients), and neurologic disease (186 patients). One cause did not exclude others. It was an “ICU with a partial liberalization of visiting policies” where families were allowed to visit their relatives every day from 1:00 pm to 9:00 pm, and medical staff gave them daily updates about their clinical situation. During the period of the study, the staff was composed of 84 people: 19 consultants, 15 residents, 38 nurses, and 12 healthcare assistants (HCAs).

Data collection was carried out from 15 October 2020 to 31 October 2020, while the remaining period was dedicated to data processing.

This study should be considered a step in a more complex process: the main objective is to highlight the need for structured CEC in ICUs and to describe the most effective strategies to be implemented in CES, both in terms of operating methods and in terms of core topics. This study therefore presents the following objectives:

1. To investigate which ethical issues are most strongly felt as important by the healthcare workers in the ICU
2. To explore the possible operating procedures to be adopted by clinical ethicists in the ICU
3. To analyze the differences between the subgroups of subjects studied in relation to the answers provided

The study concerned the HCPs in the ICU who had been employed for at least 6 months, and who belonged to the following categories: consultants, residents, nurses, and healthcare assistants (HCAs). All of these HCPs play a role in the decision-making process concerning core aspects of clinical ethics: for example, consultants investigate the clinical needs, residents help them to do it (and in doing so improve their skills), nurses are the main “patient interpreters,” and HCAs usually do not take part in the decision-making process concerning core aspects of clinical ethics, but they know the patients and their health and socio-psychological situation (in this way, they can help medical staff to detect potential ethical issues).

In order to carry out the study, an ad hoc questionnaire was used, which was prepared through a discussion process among the authors. The anonymous questionnaire consisted of 12 multiple-choice questions.

The writing of the questionnaire began in February 2020 and was suspended between March 2020 and August 2020 due to the difficulties related to the COVID-19 pandemic. The questionnaire was divided into two parts. The first five questions concerned the socio-demographic and professional characteristics of the respondents. The following seven questions investigated the ethical doubts most encountered by ICU staff members in their daily work in the ICU (question 6) and their expectations regarding the possible presence of a clinical ethicist in these wards (questions 7-12) (see Additional file 1). The questionnaire, submitted to ICU staff members in a paper format, was distributed to all HCPs in the ICU according to the inclusion and exclusion criteria. The study was approved by the Ethics Committee of Brescia in the session on 13 October 2020 (NP 4422).

### Statistical analysis

The data collected were transcribed onto a Microsoft Excel^®^ database. The dataset was analyzed through the usual univariate analyzes; the results were expressed as mode, frequency distribution, and proportion. For the third objective, Pearson’s chi-square test was used with a level of statistical significance set at *p* < 0.05. The number of subjects that were considered a priori as recruitable was about 70–80. Since this was a pilot study, there was no main expected result on which to calculate the sample size. Since this was also a descriptive study, the primary interest was not inferential but rather that of having a fair estimate of the proportions and response frequencies for each question. It was therefore decided that the expected number of 70-80 questionnaires was sufficient in this regard.

## Results

The questionnaire was proposed to all 84 HCPs who worked in that ICU, and 73 of them (87%) returned the completed questionnaire: 18 consultants, 13 residents, 32 nurses, and 10 HCAs.

Most of the HCPs were women (66%) and were aged between 30 and 40 years (55%). HCPs were consultants (24%), fellows (18%), nurses (44%), and HCAs (14%). Sixty-six percent of respondents had fewer than 11 years of ICU work experience. Concerning the religious sphere, a large majority of the respondents claimed to practice a form of religious worship (70%) (Table 1).

**Table 1:** Sociodemographic and professional characteristics of respondents (questions 1–5)

	*N* (%)
*Tot. answers*	73 (100)
*Profession*	
Consultants	18 (24)
Residents	13 (18)
Nurses	32 (44)
HCAs	10 (14)
*Age*	
< 30 yrs	5 (7)
30–40 yrs	39 (53)
41–50 yrs	24 (33)
51–60 yrs	2 (3)
> 60 yrs	3 (4)
*Female gender*	48 (66)
*Religion*	
Believer	51 (70)
Non-believer	20 (27)
No answer	2 (3)
*Years of work in ICU*	
< 5 yrs	35 (48)
5–10 yrs	13 (18)
11–20 yrs	17 (23)
21–30 yrs	6 (8)
>30 yrs	2 (3)

*HCAs* healthcare assistants, *Yrs* years, *ICU* intensive care unit

The issues that ICU staff members perceived as relevant ethical dilemmas were the donation of organs in circulatory death (37 HCPs, 51%), followed by the different diagnostic, therapeutic, and prognostic opinions among colleagues (30 HCPs, 41%) and by the decisions regarding admitting a patient to the ICU unit considering the limited resources available (29 HCPs, 40%). Most of the respondents (51, 70%) considered the clinical ethicist as a “facilitator” who analyzes the different positions existing among the members of the healthcare team and who works to find shared resolutions to ethical dilemmas, and 35 respondents (48%) believed that the clinical ethicist should be consulted especially in training or educational courses, contributing to drafting guidelines and recommendations.

Most of the respondents (53, 75%) considered that clinical ethics consultation should be provided “as needed.” Seventy-seven percent (56 HCPs) thought that all those involved in the care process (team, family members and patient) should interface with the clinical ethicist. According to the answers received, the respondents consider the presence of the clinical ethicist to be important in dealing with “end of life” issues (55, 75%), in the choice for ICU admission (31, 42%), and during family conferences (30, 41%). Most of the HCPs (65, 89%) had thought previously about the need for ethics consultation, and all respondents considered it useful. Most of the respondents (54, 74%) also considered it a priority (Table 2).

**Table 2:** Answers to questions 6–12

	Total	*n* (%)
*6.* What are the main ethical doubts or difficulties you encounter in your daily work in the intensive care unit?	192	
a. Withdrawing treatments in end-of-life situations		23 (12)
b. Donation of organs after circulatory death		38 (20)
c. PEG/tracheostomy in patients suffering from chronic-degenerative diseases (e.g., ALS)		6 (3)
d. The “limited” approaches [“skill-limited,” “time-limited,” and “event-limited”]		12 (6)
e. Deep palliative sedation at the end of life		7 (4)
f. The relationship between clinical and research activities		11 (6)
g. The advance directives		8 (4)
h. The communication of “bad news”		16 (8)
i. The conflicts between the care team and the family or between the members of the family itself		9 (4)
j. The differences in diagnostic, therapeutic and prognostic opinions among colleagues		32 (17)
k. The decision to admit a patient to the ICU considering that the resources are not infinite		30 (16)
*7.* What are your expectations regarding the intervention of the clinical ethicist?	120	
a. The clinical ethicist should be a “facilitator,” helping to analyze the different positions existing among the members of the health team and to find shared solutions to ethical dilemmas		53 (44)
b. The clinical ethicist, when asked, must analyze and offer solutions to the ethical dilemma that a clinical case presents		16 (13)
c. The clinical ethicist must analyze the different possibilities of resolving the case, but without necessarily reaching a single and definitive solution		16 (13)
d. The clinical ethicist can be consulted especially in training/refresher courses for the department in reference to specific clinical cases/contributing to the drafting of guidelines/recommendations		36 (30)
*8.* At what moment could the presence of the clinical ethicist in the ward be considered most effective?	73	
a. They should be called whenever the need arises		54 (74)
b. It is better to agree on his/her presence on a set day		1 (1)
c. The best time would be during the daily rounds		14 (20)
d. It would be most useful at specific times of the day		4 (5)
*9.* With whom should the clinical ethicist interface?	73	
a. With the medical coordinator and the director of the Dept.		0 (0)
b. With the doctor requesting the consultation		1 (1)
c. With all the professionals involved in the care of that patient		16 (22)
d. With the healthcare team and, if necessary, also with family members and, where possible, with the patient		56 (77)
*10.* At what moment could the clinical ethicist be of most help?	135	
a. Mainly in “recommendation for ICU admission”		32 (24)
b. Mainly in “end of life” issues		57 (42)
c. During the rounds		15 (11)
d. During the interview with family members		30 (22)
e. In follow-up visits		1 (1)
*11.* Before this questionnaire was submitted to you, did you ever think about the need for ethical counselling in the ICU?	73	
a. Often		29 (40)
b. Sometimes		36 (49)
c. Almost never		3 (4)
d. Never		5 (7)
*12.* At the end of this questionnaire, it is your opinion that the activation of a clinical ethics service for intensive care is:	73	
a. Useless		0 (0)
b. Useful, but not a priority for this ICU, which has more urgent needs		19 (26)
c. Very useful, representing a priority on par with “classic” clinical priorities		54 (74)

*PEG* percutaneous endoscopic gastrostomy, *ALS* amyotrophic lateral sclerosis, *ICU* intensive care unit

Table 3 showed the prevalence of the answers given by the HCPs to question no. 6 of the questionnaire in relation to the distribution of the same ICU staff members in the categories identified by answer no. 1.

**Table 3:** Answers to question 6 divided by professional profile

	Consultants, *n* (%)	Residents, *n* (%)	Nurses, *n* (%)	Hcas, *n* (%)
*6.* What are the main ethical doubts or difficulties you encounter in your daily work in the intensive care unit?	tot: 50 (100)	tot: 35 (100)	tot: 82 (100)	tot: 25 (100)
a. Withdrawing treatments in end-of-life situations	6 (12)	6 (17)	10 (12)	1 (4)
b. Removal of organs with circulatory death	11 (22)	0 (0)	22 (27)	5 (20)
c. PEG/tracheostomy in patients suffering from chronic-degenerative diseases (e.g., ALS)	1 (2)	1 (3)	4 (5)	0 (0)
d. The “limited” approaches [“skill-limited,” “time-limited,” and “event-limited”	4 (8)	5 (14)	2 (2)	1 (4)
e. Deep palliative sedation at the end of life	1 (2)	0 (0)	4 (5)	2 (8)
f. The relationship between clinical and research activities	1 (2)	1 (3)	7 (9)	2 (8)
g. The advance directives	1 (2)	2 (6)	3 (4)	2 (8)
h. The communication of “bad news”	6 (12)	5 (14)	5 (6)	0 (0)
i. The conflicts between the care team and the family or between the members of the family itself	3 (6)	4 (11)	1 (1)	1 (4)
j. The differences in diagnostic, therapeutic, and prognostic opinions among colleagues	4 (8)	3 (9)	18 (22)	7 (28)
k. The decision to admit a patient to the ICU considering that the resources are not infinite	12 (24)	8 (23)	6 (7)	4 (16)

*PEG* percutaneous endoscopic gastrostomy, *ALS* amyotrophic lateral sclerosis, *ICU* intensive care unit

Finally, the only statistically significant difference between the subgroups was identified in Table 1 according to profession, age, gender, religion, and years of work in ICU as regards the answers to question no. 6 (“What are the main ethical doubts or difficulties you encounter in your daily work in the intensive care unit?”). They indicated that the theme of the relationship between clinical activity and research activity is mostly felt by nurses and less by other HCPs (Table 3).

The detailed results of the statistical analysis by subgroups regarding the answers to questions 1–6 are shown in Table 3.

The results of the survey present a very complex profile, but on the whole, the answers to the questionnaire indicate that the need for ethics consultation in the ICU is urgent, the institutionalization of the clinical ethics service is seen as useful and should be a priority, and the issues on which the HCPs would like ethics consultation are various. All of these belong to “end of life” topics.

## Discussion

In this survey, the need for CEC perceived by the HCPs of an ICU was investigated. The questionnaire used in the study was proposed to workers in a large general Italian ICU (this ICU has a number of beds which is higher than that of most Italian ICUs), which is part of a multi-specialist hospital of the highest clinical-scientific level [18]. More than half of the respondents defined themselves as “believers” (although the degree of their faith and their specific affiliations were not investigated in detail), but this did not result in any difference in the distribution of the answers to the specific questions related to ethics consultation (questions 6–12).

Most ICU staff members were of the opinion that the clinical ethicist should help all the involved parties in a clinical case in making a shared ethical decision but should not be a “final judge” [5] of ethical disputes in a clinical setting. A considerable number of ICU staff members identified the clinical ethicist as a valid collaborator in the field of training, updating, and drafting operating procedures, testifying to the fact that the staff feel an important need for training in this area.

In reference to the first objective of the study, the “end of life” issues, as prior studies suggested, were those most deeply felt. Regarding the intervention of the clinical ethicist in ICUs, it was deemed most useful whenever there is a specific need [19–21].

Some examples are as follows: the compelling theme of the choice of patients to be treated, when the disproportion between needs and resources becomes unsustainable [22]; the ethical dilemmas regarding the withdrawal of life-sustaining treatments [23] (in some cases, the prolonging of a “biology” does not mean the prolonging of a “biography”); and the donation of organs for transplant, above all after circulatory death [24–27].

These are just some of the emerging ethical issues in the ICUs the solutions for which can hardly be found within the treatment team alone, made up of specialists and super-specialists who are trained in the medical field, but not in the ethical one. This is one of the reasons why the ICU unit represents an interesting context in which to apply CEC [28, 29].

Moreover, the working method must also be defined, since some principles (such as that of sharing decisions within the team and between the team and the patient/family) are widely shared, but those regarding the methods of conducting a CEC are less shared in this particular care setting [30].

The answers to the last two questions of the questionnaire (“Before this questionnaire was submitted to you, did you ever think about the need for ethical counselling in the ICU?” and “At the end of this questionnaire, it is your opinion that the activation of a clinical ethics service for intensive care is…?”) are more informative if they are read together.

In general, the subject of “clinical ethics” was considered a priority by the HCPs of this ICU, although a small number of respondents did not consider it to be so.

Regarding the second aim of the study, the most widespread opinion was that the ethics consultant should not only interface with the team as a whole, but also with patients and families, a factor that could express the propensity to involve the entire universe that revolves around the patient in the treatment (and decision-making) process [30, 31]. This is much easier to achieve in an “ICU with a partial liberalization of visiting policies”.

Regarding the third objective of the study (differences between the various categories of respondents regarding answers given), a general homogeneity can be observed. Neither age, nor sex, nor seniority of service, nor whether they are believers represent a reason for the polarization of the responses. On the contrary, different professional figures had different perceptions: in particular, the issue of the donation of organs after circulatory death was central for nurses, as was the issue of differences in diagnostic, therapeutic, and prognostic opinions, while for the doctors, the topic of indication for admission to the ICU was more binding. A common denominator in all these differences of opinion was that all would benefit from training, which helps professionals to better understand the technical phenomena and the dialogical methods involved in the decision-making process. And in this educational process, as indicated both by respondents to this survey and in previous studies, the clinical ethicist should play an important role [7, 17, 28, 29].

### Limitations of the study

This study has some limitations.

Firstly, the study was performed before the activation of the ethics service. Respondents answered questions about the usefulness of a service they had not yet experienced. We also focused only on HCPs and did not investigate the patients’ and family members’ experience of ethics services.

Secondly, the participants’ religious identity was not fully investigated, particularly regarding two aspects: whether the HCP was a “believer” or not and the degree of their religiosity. Thirdly, we did not conduct a “clarity test” before administering the questionnaire to HCPs.

### Future goals

A CEC who interfaces with the ICU HCPs should act both on call and as needed. The ethicist should not be a neutral “mediator.” He/she should work to improve both the decision-making process and the outcome of the process, given the moral responsibility linked to said process and the complex clinical choices to be made [32]. These choices often involve ethical dilemmas which cannot be “categorized” in a pluralistic cultural context.

Our hope is that the clinical ethicist will be able to work in an ICU together with the healthcare team by providing specialistic advice which is in line with that offered by the other specialistic consultations carried out in the hospital setting. Through specific methods and approaches, the ethics consultant is called upon to analyze and facilitate the resolution of conflicts, taking into consideration all of the stakeholders involved. The CEC thus clarifies those ethical questions which arise so that a choice may be made, together with the HCPs, regarding the most appropriate treatment path in a pluralistic and multidisciplinary medical context [33].

## Conclusions

The need for ethical advice is strongly perceived by ICU staff members. Specifically, ethics consultation should primarily focus on “end of life” issues and the figure of the clinical ethicist should become an integral part of healthcare teams in Italy.

## Supplementary Information

**Additional file 1.** Questionnaire described and cited in the main manuscript.

## Data Availability

The datasets used and analyzed during the current study are available from the corresponding author on reasonable request.

## Abbreviations

CEC: Clinical ethics consultation
CES: Clinical ethics service
HCAs: Healthcare assistants
HCPs: Healthcare professionals
ICU: Intensive care unit
