# Supplementary material for: The need for clinical ethics consultation: a monocentric observational survey study in the intensive care unit (Consul.E.T.I. study)
Source: J Anesth Analg Crit Care. 2022 Sep 14;2:40. doi: 10.1186/s44158-022-00069-0 (PMC9473475; doi:10.1186/s44158-022-00069-0)
Supplement: Supplementary file 1 — Additional file 1. Questionnaire described and cited in the main manuscript. [file 44158_2022_69_MOESM1_ESM.pdf]

Additional file 1: Questionnaire

The questionnaire consists of 12 questions for which only one answer is required, unless otherwise indicated.

1- What is your profession?

□ Consultant

□ Resident

□ Nurse

□ Health Care Assistant

2- Age:

□ <30 years

□ 30-40 years

□ 41-50 years

□ 51-60 years

□> 60 years

3- Gender:

□ M

□ F

4- From a religious / spiritual point of view, do you consider yourself:

□ believer

□ not believer

5- Years of work experience in the ICU:

□ <5

□ 5-10

□ 11-20

□ 21-30

□ >30

6- What are the main ethical doubts or difficulties you encounter in your daily work in the Intensive Care Unit?

(You may select up to 3 answers)

□ withdrawing treatments in end-of-life situations

□ donation of organs after circulatory death

□ PEG / Tracheostomy in patients suffering from chronic-degenerative diseases (e.g. ALS)

□ the “limited” approaches [“skill-limited” (for example "no indications for catecholamines, CVVH, etc.”), “time-limited” (for example “maximal therapy for 48 hours, then in the absence of response de-escalation and palliation”) and “event-limited” (for example “we will continue with maximal therapy, but if a new septic shock occurs, we will stop”)]

□ deep palliative sedation at the end of life

□ the relationship between clinical and research activities

□ the Advance Directives

□ the communication of “bad news”

□ the conflicts between the care team and the family or between the members of the family itself

□ the differences in diagnostic, therapeutic and prognostic opinions among colleagues

□ the decision to admit a patient to the ICU considering that the resources are not infinite

7- What are your expectations regarding the intervention of the clinical ethicist? (You may select up to 2 answers)

□ the clinical ethicist should be a “facilitator”, helping to analyze the different positions existing among the members of the health team and to find shared solutions to ethical dilemmas

□ the clinical ethicist, when asked, must analyze and offer solutions to the ethical dilemma that a clinical case presents

□ the clinical ethicist must analyze the different possibilities of resolving the case, but without necessarily reaching a single and definitive solution

□ the clinical ethicist can be consulted especially in training / refresher courses for the department in reference to specific clinical cases/ contributing to the drafting of guidelines / recommendations

8- At what moment could the presence of the clinical ethicist in the ward be considered most effective?

□ they should be called whenever the need arises

□ it is better to agree on his/her presence on a set day

□ the best time would be during the daily rounds

□ it would be most useful at specific times of the day (for example ___________)

9- With whom should the clinical ethicist interface?

□ with the medical coordinator and the director of the ICU.

□ with the doctor requesting the consultation

□ with all the professionals involved in the care of that patient

□ with the health care team and, if necessary, also with family members, and where possible, with the patient

10- At what moment could the clinical ethicist be of most help?

(You may select up to 2 answers)

□ mainly in "recommendation forICU admission"

□ mainly in "end of life" issues

□ during the rounds

□ during the interview with family members

□ in follow-up visits

11- Before this questionnaire was submitted to you, did you ever think about the need for ethical counselling in the ICU?

□ often

□ sometimes

□ almost never

□ never

12- At the end of this questionnaire, it is your opinion that the activation of a Clinical Ethics Service for Intensive Care is:

□ useless

□ useful, but not a priority for this ICU, which has more urgent needs

□ very useful, representing a priority on par with "classic" clinical priorities

ICU: Intensive Care Unit; PEG: Percutaneous endoscopic gastrostomy; ALS: Amyotrophic lateral sclerosis; CVVH: Continuous veno-venous haemofiltration.
